# Patients with first-episode psychosis in northern Taiwan: neurocognitive performance and niacin response profile in comparison with schizophrenia patients of different familial loadings and relationship with clinical features

**DOI:** 10.1186/s12888-024-05598-2

**Published:** 2024-02-22

**Authors:** Shun-Chun Yu, Tzung–Jeng Hwang, Chih-Min Liu, Hung-Yu Chan, Chian-Jue Kuo, Tsung-Tsair Yang, Jen-Pang Wang, Chen-Chung Liu, Ming H. Hsieh, Yi-Ting Lin, Yi-Ling Chien, Po-Hsiu Kuo, Ya-Wen Shih, Sung-Liang Yu, Hsuan-Yu Chen, Wei J. Chen

**Affiliations:** 1 Institute of Epidemiology and Preventive Medicine, College of Public Health, National Taiwan University, Taipei, Taiwan; 2 Centers for Genomic and Precision Medicine, National Taiwan University, Taipei, Taiwan; 3 Department of Psychiatry, National Taiwan University Hospital and College of Medicine, Taipei, Taiwan; 4 Neurobiology and Cognitive Science Center, National Taiwan University, Taipei, Taiwan; 5 Taoyuan Psychiatric Center, Taipei, Taiwan; 6 Taipei City Psychiatric Center, Taipei City Hospital, Taipei, Taiwan; 7 Department of Social Psychology, Shih Hsin University, Taipei, Taiwan; 8 Bethel Psychiatric Clinic, Taipei, Taiwan; 9 Department of Clinical Laboratory Sciences and Medical Biotechnology, College of Medicine, National Taiwan University, Taipei, Taiwan; 10 Institute of Statistical Science, Academia Sinica, Taipei, Taiwan; 11 Center for Neuropsychiatric Research, National Health Research Institutes, Zhunan, Miaoli County Taiwan

**Keywords:** First-episode psychosis, Schizophrenia, Sustained attention, Executive function, Niacin skin test

## Abstract

**Background:**

Examining patients with first-episode psychosis (FEP) provides opportunities to better understand the mechanism underlying these illnesses. By incorporating quantitative measures in FEP patients, we aimed to (1) determine the baseline distribution of clinical features; (2) examine the impairment magnitude of the quantitative measures by comparing with external controls and then the counterparts of schizophrenia patients of different familial loadings; and (3) evaluate whether these quantitative measures were associated with the baseline clinical features.

**Methods:**

Patients with FEP were recruited from one medical center, two regional psychiatric centers, and two private clinics in northern Taiwan with clinical features rated using the Positive and Negative Syndrome Scale (PANSS) and Personal and Social Performance (PSP) scale. Quantitative measurements included the Continuous Performance Test (CPT), Wisconsin Card Sorting Test (WCST), niacin response abnormality (NRA), and minor physical anomalies and craniofacial features (MPAs). To evaluate the relative performance of the quantitative measures in our FEP patients, four external comparison groups from previous studies were used, including three independent healthy controls for the CPT, WCST, and NRA, respectively, and one group of treatment-resistant schizophrenia patients for the MPAs. Additionally, patients from simplex families and patients from multiplex families were used to assess the magnitude of FEP patients’ impairment on the CPT, WCST, and NRA.

**Results:**

Among the 80 patients with FEP recruited in this study (58% female, mean age = 25.6 years, mean duration of untreated psychosis = 132 days), the clinical severity was mild to moderate (mean PANSS score = 67.3; mean PSP score = 61.8). Patients exhibited both neurocognitive and niacin response impairments (mean Z-scores: −1.24 for NRA, − 1.06 for undegraded d', − 0.70 for degraded d', − 0.32 for categories achieved, and 0.44 for perseverative errors) but did not show MPAs indicative of treatment resistance. Among these quantitative measures, three of the four neurocognitive indices were correlated with the baseline clinical features, whereas NRA did not show such correlation.

**Conclusions:**

This FEP study of Taiwanese patients revealed the presence of neurocognitive performance and niacin response and their different relationships with clinical features, rendering this sample useful for future follow-up and incorporation of multiomics investigation.

**Supplementary Information:**

The online version contains supplementary material available at 10.1186/s12888-024-05598-2.

## Introduction

Despite progress in treatment over the past fifty years, schizophrenia and other forms of psychotic disorders remain highly debilitating in the first decade of the 21st century and confer a heavy burden on individuals, health care systems, and society in general [1]. Since the early 1990s, many studies recruiting people with first-episode psychosis (FEP) have been initiated to explore the nature and course of the psychotic illness [2], and a substantial proportion of FEP patients have poor long-term functional outcomes, including impairments in cognitive or social functioning [3]. Among studies that employed a systematic ascertainment, the diagnostic breadth of patients with FEP includes three main nodes, i.e., schizophrenia spectrum psychosis, bipolar disorder and major depressive disorder with psychotic features, as well as psychotic disorder not otherwise specified [4, 5]. Furthermore, a patient’s diagnosis might change during the disease course [6], indicating that certain underlying vulnerabilities among various psychotic illnesses might be shared at an early stage.

Using the most common form of psychosis, schizophrenia, as an example, it has a heritability greater than 80% [7], and a variety of clinical, neuropsychological, and biological measures have been proposed as diagnostic or theragnostic markers [8]. Among them, the endophenotype approach seeks measures that may reflect underlying traits with increased genetic susceptibility to the target disease or phenotype, i.e., the prevalence of an endophenotype being higher not only in patients with the target disease but also in nonpsychotic relatives of the patients than in healthy controls, and greater familial loading of the target disease being associated with more impairment in the trait [9]. As pointed out in a review [10], the relations of a variety of endophenotypes to the underlying susceptibility genes and final onset of the disease could be explained using the sufficient-component causal model [11, 12], with each endophenotype representing a separate sufficient-component of the cause. Several candidate endophenotypes of schizophrenia have been proposed, including impairment on the Continuous Performance Test (CPT), impairment on the Wisconsin Card Sorting Test (WCST), and niacin response abnormality (NRA) [10, 13]. In addition, a previous study of FEP cohort found that not only patients with non-affective psychosis but also patients with affective psychosis differed significantly from controls on minor physical anomalies and craniofacial features (MPAs) [14]. Following this line of research, a later study examining MPAs in schizophrenia patients found that certain features were associated with treatment resistance [15].

Despite the abundance of literature on FEP, many studies have been conducted in Caucasian populations and only a few have been conducted in Asian populations; e.g., only 6 out of 58 studies included in a recent meta-analysis on hospitalization following FEP were from Asia [16]. To date, limited numbers of Asian FEP studies have tended to only focus on either neurocognitive measures [17–20] or the NRA [21–23]. Besides, nonpsychotic relatives of multiplex schizophrenia families showed greater cognitive impairment than those of simplex ones, indicating that a higher familial loading of schizophrenia was associated with a poorer neurocognitive performance [24–28]. Whether those quantitative measures could reflect differential vulnerabilities to psychosis with different familial loadings warrants further investigation.

To fill these gaps in the literature, we established a cohort of patients with FEP in northern Taiwan with several quantitative measures, including the CPT, WCST, NRA, and MPAs. We aimed to (1) determine the baseline distribution of clinical features; (2) examine the impairment magnitude of the quantitative measures by comparing with external controls and then the counterparts of schizophrenia patients of different familial loadings; and (3) evaluate whether these quantitative measures were associated with the baseline clinical features of FEP patients.

## Methods

### Participants

In a prospective cohort study of patients with FEP, participants were recruited from both the outpatient clinics and inpatient psychiatric wards of the participating hospitals (one medical center and two regional psychiatric centers) and clinics in northern Taiwan, including National Taiwan University Hospital, Taipei Psychiatric Center, Taoyuan Psychiatric Center, and two private clinics. Inclusion criteria were as follows: aged 15–45 years, having an ethnicity of Taiwanese Han, experiencing FEP (the first onset of psychotic symptoms within one year), and being antipsychotic-naïve or minimally treated (< 3 month of treatment with any psychotic medication). The diagnoses were based on the Diagnosis and Statistical Manual of Mental Disorders, Fifth Edition (DSM-5) and determined by psychiatrists in the consensus meeting. Patients with substance-induced psychosis, organic brain disorder, or mental retardation were excluded. For participants aged < 20 years, both the participant and one parent/legal guardian provided written informed consent after a complete description of the study; otherwise, only the participant provided the written informed consent. This study was approved by the Research Ethics Committee of each participating hospital (National Taiwan University Hospital: 20150203RINC; Taipei City Hospital: TCHIRB-10,501,107; and Taoyuan Psychiatric Center: B20151222).

To evaluate the relative performance of the quantitative measures in our FEP patients, four external comparison groups from previous studies were used, including a community sample of 345 individuals [29] for the CPT, a group of 440 healthy controls [28] for the WCST, a group of 94 healthy controls [30] for the NRA, and a group of 108 treatment-resistant schizophrenia patients [15] for the MPAs.

Additionally, the magnitude of impairment on the CPT, WCST, and NRA in FEP patients were further compared with the counterparts from two external samples of schizophrenia patients with different familial loadings, including patients from simplex families (1649 for CPT and WCST, and 1866 for NRA), i.e., only one affected person in a nuclear family [31] and patients from multiplex families (1314 for CPT and WCST, and 176 for NRA), i.e., co-affected siblings in a nuclear family [32].

### Measurement

Patients were interviewed using the Chinese Version of the Diagnostic Interview for Genetic Studies (DIGS) [33, 34] by well-trained research assistants at baseline. Antipsychotics dosage at baseline were collected and converted to chlorpromazine equivalents [35]. In addition, each patient received periodic evaluations of a variety of domains, including (1) clinical symptoms using the Positive and Negative Syndrome Scale (PANSS) at baseline and at the 1st, 3rd, 6th, 12th, 18th and 24th months; (2) social functioning using the Personal and Social Performance (PSP) scale at baseline and at the 6th month; (3) measurements on the endophenotype for schizophrenia, including the CPT, WCST, and NRA at baseline and at the 6th month; and (4) MPAs at baseline and at the 24th month. Among them, the PANSS and PSP scale were rated by psychiatrists, and the remaining evaluations were conducted by research assistants. All the participating psychiatrists and research assistants had received relevant training on the use of each instrument, as briefly described below.

(1) The Chinese version of the PANSS [36, 37] has been shown to have good interrater reliability (with intraclass correlation coefficients ranging from 0.64 to 0.96) [38]. The PANSS consists of 30 items, each with a 7-point rating scale, and it is classified into the positive symptom subscale (7 items), the negative symptom subscale (7 items), and the general psychopathology subscale (16 items).

(2) The PSP scale [39] is a 100-point rating scale to judge the degree of difficulties in four specific aspects of social functioning: (1) socially useful activities, (2) personal and social relationships, (3) self-care, and (4) disturbing and aggressive behaviors. A lower score indicates poorer personal and social performance. The PSP scale has high reliability and validity in patients with schizophrenia in both acute and stable stages [40, 41]. The Chinese version of the PSP scale was found to have a reliability of 0.91 [40].

(3) The procedure and reliability of data obtained from the CPT [42] for use in this study are described in more detail elsewhere [29]. The sensitivity index (d′) of CPT performance was derived from the difference between the normal deviation of the hit rate (probability of response to target trials) and that of false alarm.

(4) The WCST was used to assess patients’ executive function [43, 44], and two performance indices, perseverative errors and categories achieved, were used for subsequent analyses.

(5) The NRA was measured by applying absorbent paper with equal volumes of three concentrations of aqueous methyl nicotinate (0.1, 0.01, and 0.001 M) as well as a blank negative control to each subject’s forearm skin for 5 min. The paper was removed to rate the flush response at 5, 10, and 15 min using a 4-point scale ranging from 0 to 3, which was shown to have excellent interrater reliability [30]. Since the flush response to 0.001 M exhibited a poor ability to discriminate between patients with schizophrenia and normal controls [30], we used the volumetric niacin response (VNR) [45] to summarize the flush score over the three time points for concentrations of 0.1 M and 0.01 M.

(6) The MPAs measured in this study, including facial width, lower facial height, and mouth score, were conducted following the procedures in a previous study [15]. Briefly, a scale was developed based on previous studies to assess both qualitatively measured MPAs (e.g., rated as the presence or absence of 4 morphological anomalies in mouth) and quantitatively measured craniofacial features using calipers, tapes and protractors by following the standardized methods used in anthropometric measurements.

### Statistical analysis

Each neurocognitive measure and NRA was transformed into a Z-score by standardizing against an external comparison group as follows: (a) CPT performance raw scores were standardized with adjustments for sex, age and education against a community sample of 345 individuals [29]; (b) WCST performance raw scores were standardized with adjustments for sex, age and education against a group of 440 healthy controls [28]; and (c) the VNR was standardized against a group of 94 healthy controls [30].

To evaluate whether the impairment was less severe in FEP patients than in schizophrenia patients of different familial loadings, the Z-scores of individual endophenotype measures were compared to the counterparts of schizophrenia patients from both simplex and multiplex families as follows: (a) the adjusted Z-scores of both the CPT and WCST of 1649 schizophrenia patients from simplex families [31] and 1314 schizophrenia patients from multiplex families [32]; and (b) the Z-scores of the VNR of 1866 schizophrenia patients from simplex families [31] and 176 schizophrenia patients from multiplex families [32]. The adjusted Z-scores were calculated using the identical external comparison group as that of FEP patients.

Additionally, the scores of qualitatively and quantitatively measured MPAs were standardized against a group of 108 treatment-resistant schizophrenia patients [15].

We tested the correlation between baseline measures and clinical features using Spearman’s correlation. We also conducted multivariable linear regression analysis of baseline measures on clinical features with adjustment for potential confounders. However, if a covariate was found to be a collider [46], i.e., a variable that is affected by both exposure and outcome variables in a causal graph, we would not include it in the final model [47]. All statistical analyses were performed using R language, version 4.0.2 [48]. All tests were two-tailed, and the significance level was 0.05. Bonferroni correction for multiple testing was adopted when evaluating the correlation of each baseline measure with three variables of clinical features.

## Results

From January 2016 to May 2019, 135 patients met the inclusion criteria, and 80 were successfully enrolled. Among 80 participants, most were female (58%), whom had an educational level of college or higher (63%), and were recruited from outpatient clinics (59%), especially those in hospitals (34%), but only a small proportion had a family history (11%) or were current smokers (9%) (Table 1). The mean duration of untreated psychosis (DUP) was 132 days (SD: 136.8; median: 71). The most common diagnoses were schizophrenia (45%), followed by other nonaffective psychosis (41%) and affective psychosis (14%). When inpatient and outpatient participants were compared, there were no significant differences except a higher proportion of schizophrenia among inpatients than outpatients (*p* = 0.02; Supplementary Table S1).

**Table 1:** Demographic characteristics, source of recruitment, and baseline diagnosis of patients with first-episode psychosis recruited in northern Taiwan from 2016–2019

Characteristics	Baseline (*n* = 80)
Sex, n (%)	
Male	34 (43%)
Female	46 (58%)
Age (years), mean (SD)	25.6 (5.0)
Duration of untreated psychosis (days), mean (SD)	132 (136.8)
Chlorpromazine equivalents, mean (SD)	196 (149.8)
Family history, n (%)	9 (11%)
Current smoking, n (%)	7 (9%)
Educational level^a^, n (%)	
≤ Junior high	4 (5%)
Senior high	22 (28%)
≥ College	50 (63%)
Recruitment source, n (%)	
Inpatients	33 (41%)
Outpatients	47 (59%)
Hospital	34
Private clinics	13
Diagnosis, n (%)	
Schizophrenia	36 (45%)
Other nonaffective psychosis	33 (41%)
Schizotypal personality disorder	2
Delusional disorder	4
Brief psychotic disorder	6
Schizoaffective disorder	7
Other specified schizophrenia spectrum and other psychotic disorder	2
Unspecified schizophrenia spectrum and other psychotic disorder	12
Affective psychoses	11 (14%)

^a^Missing data in 4 patients

Since the performance of the FEP patients were standardized against different comparison groups and their respective Z-scores were then compared with the counterparts of schizophrenia patients with different familial loadings, the sex and age distribution of these external groups are shown in Table 2.

**Table 2:** Sample characteristics of external comparison groups

	External comparison groups for standardization					Schizophrenia patients with different familial loadings			
	CPT	WCST	NR	MPAs		CPT and WCST		NRA	
Type	Community sample	Healthy controls	Healthy controls	Treatment-resistant schizophrenia patients		Simplex families	Multiplex families	Simplex families	Multiplex families
Sample size	345	440	94	108		1649	1314	1866	176
Age, mean (SD)	41.3 (13.0)	39.9 (15.7)	33.2 (10.2)	44.9 (9.1)		35.1 (8.0)	36.4 (9.6)	35.6 (8.2)	35.2 (7.9)
Male sex, n (%)	165 (48%)	191 (43%)	45 (48%)	62 (57%)		639 (39%)	509 (31%)	1149 (62%)	121 (69%)
Reference	[29]	[28]	[30]	[15]		[31]	[32]	[31]	[32]

Abbreviations: CPT = Continuous Performance Test; WCST = Wisconsin Card Sorting Test; NR = niacin response; MPAs = minor physical anomalies and craniofacial features

Regarding baseline clinical characteristics (Table 3), the mean total PANSS score was 67.3, and the severity of the positive subscale was similar to that of the negative subscale (more details in Supplementary Figure S1). The mean PSP score was 61.8, i.e., having manifested but not marked difficulties in social functioning (i.e., a score of 61–70). In addition, outpatients had a higher mean PSP score (i.e., better social functioning) than inpatients (Supplementary Table S2). Table 3 further displays both the raw scores and Z-scores of two neurocognitive measures (CPT and WCST), VNR and MPAs. During the early disease course, the FEP patients showed poorer performance on the CPT and WCST and greater attenuation in the VNR (more details in Supplementary Figure S2) than the normal controls. Compared to schizophrenia patients with treatment resistance, FEP patients showed different characteristics (a wider facial width, shorter facial height, and lower mouth score).

**Table 3:** Baseline clinical characteristics and quantitative measures among patients with first-episode psychosis in Taiwan

Variables	Total (*N* = 80) Mean (SD)	
	Raw score	Z-score
***Clinical features***		
Positive and Negative Syndrome Scale score		
Total	67.3 (23.0)	-
Positive	16.8 (7.0)	-
Negative	15.5 (7.0)	-
General psychopathology	35.1 (11.9)	-
Personal and Social Performance score	61.8 (16.9)	-
***Baseline measures***		
Continuous Performance Test		
Undegraded d’	3.9 (0.9)	−1.06 (1.3)^a ***^
Degraded d’	3.4 (1.3)	−0.70 (1.4)^a ***^
Wisconsin Card Sorting Test		
Perseverative errors	25.1 (20.3)	0.44 (1.3)^b *^
Categories achieved	5.3 (3.1)	−0.32 (1.1)^b *^
Niacin response (NR) abnormality		
Volumetric NR for 0.1 M and 0.01 M	10.0 (3.5)	−1.25 (1.2)^c ***^
Minor physical anomalies and craniofacial features		
Facial width	13.6 (0.9)	0.69 (1.0)^d ***^
Lower facial height	6.8 (0.7)	−0.28 (1.0)^d *^
Mouth score	0.5 (0.8)	−1.04 (0.7)^d ***^

^a^ Z-score adjusted for sex, age, and education against a community sample of 345 individuals [29]

^b^ Z-score adjusted for sex, age, and education against a group of 440 healthy controls [28]

^c^ Z-score against a group of 94 healthy subjects [30]

^d^ Z-score against a group of 108 patients with treatment-resistant schizophrenia [15]

^*^p value < 0.05 and ^***^p value < 0.001 versus the comparison group

Abbreviations: NR = niacin response

Based on the Z-scores of those quantitative measures that have been suggested to be candidate endophenotypes of schizophrenia, their pairwise Spearman’s correlations among patients with FEP are displayed in Table 4. The magnitude of the correlation was large between the two indices of the same neurocognitive test (0.77 for the CPT and 0.85 for the WCST) and moderate between the indices of two neurocognitive tests (ranging from 0.35 to 0.52). Meanwhile, NRA was not correlated with any index of the two neurocognitive tests.

**Table 4:** Spearman’s correlation of baseline quantitative measures among patients with first-episode psychosis (N = 80)

Z-scores	CPT undegraded d’ r (*P* value)	CPT degraded d’ r (*P* value)	WCST −Perseverative errors^a^ r (*P* value)	WCST Categories achieved r (*P* value)	Niacin response (NR) abnormality Volumetric NR for 0.1 M and 0.01 M r (*P* value)
Continuous Performance Test (CPT)					
Undegraded d’	1.00	-	-	-	-
Degraded d’	0.77 (< 0.001)	1.00	-	-	-
Wisconsin Card Sorting Test (WCST)				-	-
−Perseverative errors^a^	0.46 (< 0.001)	0.35 (0.003)	1.00	-	-
Categories achieved	0.52 (< 0.001)	0.42 (< 0.001)	0.85 (< 0.001)	1.00	-
Niacin response (NR) abnormality					-
Volumetric NR for 0.1 M and 0.01 M	−0.01 (0.94)	0.03 (0.82)	0.01 (0.94)	0.13 (0.31)	1.00 (1.00)

^a^Transformed to a negative value to let a more negative Z-score represent a greater impairment compared to the comparison group and hence denoted as − perseverative errors

Abbreviations: CPT = Continuous Performance Test; WCST = Wisconsin Card Sorting Test; NR = niacin response

**Fig. 1:**
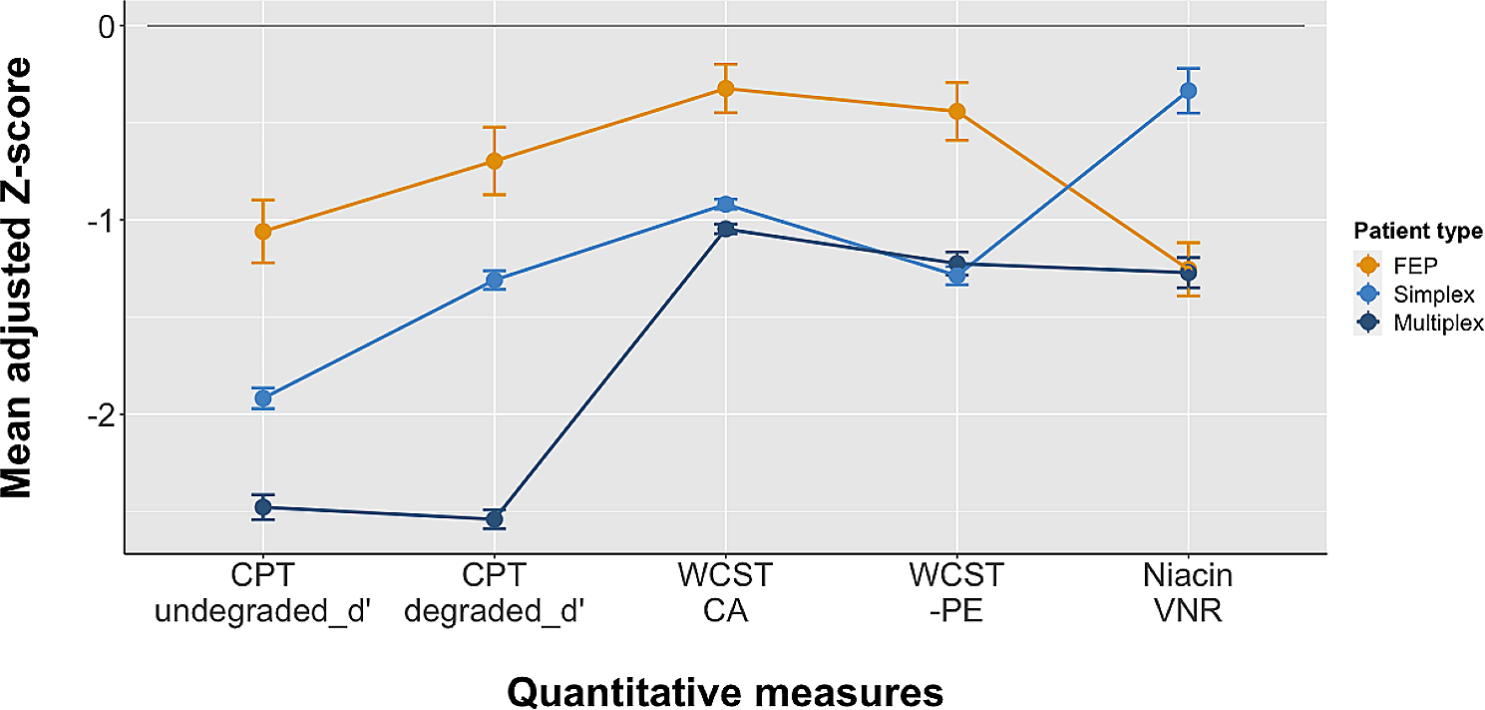
Relative deficit in terms of Z-score in Continuous Performance Test (CPT) indices, Wisconsin Card Sorting Test (WCST) indices, and attenuated niacin-induced flush response for patients with first episode psychosis versus those of schizophrenia patients from simplex families and multiplex families, respectively. The vertical bar indicates the standard error interval of the mean Z-score. CPT = Continuous Performance Test; WCST = Wisconsin Card Sorting Test; PE = perseverative errors, and–PE represents being transformed to negative value; CA = categories achieved; niacin response was based on the volumetric niacin response (VNR) for 0.1 M and 0.01 M

Figure 1 depicts the Z-scores in five quantitative measures of the FEP patients against the counterparts of two external groups of schizophrenia patients from simplex and multiplex families, respectively, with all of them being significantly lower than 0 (i.e., the mean of healthy controls’ Z-scores). Nevertheless, the distributions in the magnitude of impairment among the three groups of patients varied for different tests. For the two CPT indices (undegraded d′ and degraded d′), the impairment in the FEP patients was the smallest, followed by that in the simplex schizophrenia patients, and that in the multiplex schizophrenia patients being the greatest. For the two WCST indices, the impairment in the FEP patients remained as the smallest, whereas the counterparts of the simplex and multiplex schizophrenia patients became slightly different (categories achieved) or similar (perseverative errors). Meanwhile, for the NRA, the magnitude of attenuation in the FEP patients was greater than that in the simplex schizophrenia patients but similar to that in the multiplex schizophrenia patients. More detailed results of the group comparison using ANOVA with Tukey post hoc comparison are provided in Supplementary Table S3. To examine the robustness of the distinction among the three group of patients (FEP, simplex patients, and multiplex patients), we also conducted a multinomial logistic regression analysis using simplex patients as the reference outcome. The results turned out to be the same as those of ANOVA, i.e., the odds ratios (ORs) of FEP were significantly < 1.0 for all 5 quantitative measures whereas those for multiplex patients were significantly > 1.0 except − perseverative errors (Supplementary Table S4).

We then evaluated the relationship between the Z-scores of neurocognitive measures, VNR or MPAs and the baseline clinical features, with correction for multiple testing for three variables of clinical features (Table 5). For CPT performance, greater impairment in undegraded d’, but not degraded d’, was associated with increased symptoms in both positive and negative subscales and poorer social functioning (*p* = 0.004, < 0.001, and 0.005, respectively). For WCST performance, greater impairment in both perseverative errors and categories achieved was associated with increased symptoms in both positive (*p* = 0.006 and 0.002, respectively) and negative subscales (both *p* < 0.001) but not with PSP scores *p* = 0.06 and 0.03, respectively). For the niacin flush response, the VNR was not associated with any variables of clinical features. Similarly, none of the MPAs were associated with baseline clinical features.

**Table 5:** Spearman’s correlation of baseline measures with clinical features at baseline among patients with first-episode psychosis (N  = 80)

Z-scores	PANSS positive r (*P* value)	PANSS negative r (*P* value)	PSP r (*P* value)
Continuous Performance Test			
Undegraded d’	−0.35 (0.004)^*^	−0.50 (< 0.001)^*^	0.35 (0.005)^*^
Degraded d’	−0.13 (0.30)	−0.29 (0.02)	0.29 (0.02)
Wisconsin Card Sorting Test			
−Perseverative errors^a^	−0.33 (0.006)^*^	−0.45 (< 0.001)^*^	0.23 (0.06)
Categories achieved	−0.38 (0.002)^*^	−0.43 (< 0.001)^*^	0.27 (0.03)
Niacin response (NR) abnormality			
Volumetric NR for 0.1 M and 0.01 M	−0.26 (0.04)	−0.07 (0.60)	0.05 (0.69)
Minor physical anomalies and craniofacial features			
Facial width	0.02 (0.87)	0.12 (0.32)	0.00 (0.98)
Lower facial height	−0.01 (0.96)	0.07 (0.60)	0.08 (0.55)
Mouth score	0.03 (0.81)	0.00 (0.99)	−0.01 (0.92)

^a^Transformed to a negative value to let a more negative Z-score represent a greater impairment compared to the comparison group and hence denoted as − perseverative errors

^*^P < (0.05/3

0.016), with correction for multiple testing for variables of clinical features

Abbreviations: PANSS = Positive and Negative Syndrome Scale; PSP = Personal and Social Performance; NR = niacin response

To evaluate whether the relationship between clinical features and quantitative measures were confounded by other variables, we conducted a series of correlation analysis between potential confounders and these two groups of variables (Table S5). Antipsychotic dosage and current smoking status were found to be associated with both PANSS positive subscales and neurocognitive measures. Hence, we further conducted multivariate regression analysis to evaluate the influence of two potential confounders. Given that antipsychotic dosage was likely to be prescribed based on a comprehensive consideration of the patient’s current symptoms, which may also include cognitive functioning, antipsychotic dosage was considered as a collider. Indeed, adjusting for antipsychotic dosage at baseline led to the disappearance of the associations between PANSS positive subscales and neurocognitive measures, which might be caused by collider bias (Table S6). Therefore, antipsychotic dosage was not included in the final regression analysis. With adjustment for current smoking status, the association between PANSS positive subscales and neurocognitive measures, also shown in Table S6, were similar to the results of correlation.

## Discussion

In this study of Taiwanese patients with FEP, patients exhibited impairments in neurocognitive performance, including the CPT (undegraded d′ and degraded d′) and WCST (perseverative errors and categories achieved), as well as in the NRA, and did not have the MPAs profile characteristic of treatment-resistance. When the indices of the CPT, WCST, and NRA were standardized against external healthy comparison groups, the Z-scores of our FEP patients ranged from approximately 1 to < 0.5 standard deviations below the comparison group. Among these Z-scores, there were large (within-test) to moderate (between-test) pairwise correlations among the indices of CPT and WCST, but none between these neurocognitive indices and NRA. The magnitudes of impairment on the CPT and WCST in the FEP patients were smaller than those of two external groups of schizophrenia patients from simplex or multiplex families, whereas that on the NRA in the FEP patients was more prominent than that in the simplex schizophrenia patients but similar to that in the multiplex ones. These quantitative measures displayed differential relationships with the baseline clinical features of the FEP patients, with the NRA not correlating with clinical features, whereas three of the four neurocognitive indices did correlate. These results help shed new light on the underlying vulnerability of FEP.

The majority of our FEP participants (59%) were not hospitalized at the time of recruitment, which is comparable to the findings of a continuous decrease in the first admission rate for psychosis from 1998 to 2007 [49] as well as from 2001 to 2017 [50] in Taiwan. These FEP patients were in the early stage of their illness, with a PANSS total score-based severity level between mildly ill (58) and moderately ill (75) [51], a PSP score indicating modest difficulties in social functioning, and a DUP much shorter than the average of 387.7 days in a meta-analysis of 40 FEP studies [52]. Hence, our FEP patients provided an opportunity to examine the presence of neurocognitive performance, NRA and MPAs without being confounded by treatment or disease stage.

Regarding the neurocognitive impairment in our FEP patients, the magnitude of the mean of Z-scores for the CPT indices (− 1.06 for undegraded d′ and − 0.70 for degraded d′) were greater than those for the WCST indices (− 0.32 for categories achieved and 0.44 for perseverative errors). The presence of neurocognitive impairment in FEP patients has been replicated in two meta-analyses: one included studies up to 2013 [53], with effect sizes ranging from 0.44 to 1.56 for CPT, 0.51 to 1.86 for perseverative errors, and 0.53 to 0.98 for categories achieved; and the other included studies involving Chinese patients up to 2019 [54], with a mean Z-score of -1.33 for the CPT and − 1.04 for problem solving (i.e., the WCST). Interestingly, the estimates from more recent studies were − 1.12 for the CPT and − 0.38 for the WCST in one U.S. study [55], and − 0.92 for the CPT and − 1.14 for the WCST in one Spanish study [56]. Taken together, the magnitude of impairment on the CPT was more similar across recent studies than that of the WCST. This phenomenon is comparable with previous findings that the heritability estimates for the CPT [57] were higher than those of the WCST [28], indicating that the performance on the WCST had greater environmental contribution than the counterpart on the CPT.

Regarding FEP patients’ NRA, the mean Z-score (− 1.24) was of similar magnitude to that of the CPT undegraded d′. Previous FEP studies examining the NRA also reported its presence in Chinese [21–23] and German [58] patients, but did not provide the corresponding Z-scores.

The derivation of Z-scores for the five quantitative measures of three tests (CPT, WCST, and NRA) in the FEP patients provides us an opportunity to further explore their underlying vulnerabilities in three aspects. First, in terms of inter-correlations, the impairments on the CPT and WCST were related whereas that on NRA was not, despite their similar values of Z-score.

Second, since a greater genetic susceptibility was implicated by an increasing trend of greater magnitude of impairment in quantitative measures with greater familial loading of schizophrenia not only in patients but also in their unaffected relatives, including the indices on the CPT [25, 59, 60], WCST [28, 59, 61], and [62], we compared the Z-scores of these measures in the FEP patients to the counterparts of simplex and multiplex schizophrenia patients. Intriguingly, both the CPT and WCST displayed a pattern different from that of the NRA. The magnitudes of impairment on both the CPT and WCST were less than those of simplex schizophrenia patients, which were less than those of multiplex schizophrenia patients except perseverative errors on the WCST. These results partly extended previous findings that there is polygenic overlap between schizophrenia and neurocognitive performance [31], i.e., the polygenic architecture of susceptibility to schizophrenia modified patients’ neurocognitive performance, in simplex schizophrenia patients to FEP patients. Nevertheless, given that our FEP patients had a mixture of familial loadings (11% of them having a family history of psychiatric illness), the magnitude of impairment would be as severe as that of simplex schizophrenia patients if the impairment was merely due to genetic susceptibility. Hence, the results of less impairment on both the CPT and WCST in the FEP patients than in simplex schizophrenia patients might be further accounted for by the longer duration of illness of the schizophrenia patients in previous studies, with a mean of 12.6 years for simplex patients [32] and 14.0 years for multiplex patients [31], which were associated with a greater magnitude of impairment in schizophrenia patients [63]. In contrast, the pattern of the impairment on the NRA in the FEP patients as compared to the schizophrenia patients of different familial loadings was contradictory to the previous finding of an increasing trend in both patients and unaffected relatives from simplex families to multiplex families [62]. Since extant studies have seldom compared the NRA in FEP patients to that in schizophrenia patients with different familial loadings, further investigation is warranted to clarify whether the discrepancy is due to the variations in the rating of NRA across studies. Another possibility is that the underlying mechanism of the NRA is distinct from that underlying the schizophrenia of different familial loadings.

Third, the Z-scores of these quantitative measures were found to have different relations to the baseline clinical features, i.e., the NRA did not correlate with clinical features, whereas three of the four neurocognitive indices did so with both the positive and negative subscales of the PANSS and the CPT undegraded d′ had further correlation with the PSP score. To date, only a few FEP studies have examined this relationship via different analytic approaches toward PANSS scores, e.g., one study reported a modest correlation between categories achieved and a cognitive factor [64] and another one found a negative correlation between other cognitive functions and disorganization [65]. Under this circumstance, our findings reveal that the neurocognitive performance was correlated comprehensively with clinical features at baseline and, hence, may worsen with more severe symptoms over the course of illness, which may render the magnitude of neurocognitive impairment more similar to that of simplex schizophrenia patients.

Our findings have implications for future research. One implication is to examine the longitudinal pattern of clinical features, exemplified in cohort studies in which patients with no or mild negative symptoms had better neurocognitive performance than patients with sustained negative symptoms [63, 66]. In addition, whether these FEP patients’ MPAs were indicative of treatment resistance requires follow-up data of clinical features. Another implication is that the underlying mechanism of the niacin flush abnormality might be different from that of neurocognitive impairment. Indeed, the neurocognitive impairment in schizophrenia has been implicated as imbalanced interactions between excitatory and inhibitory neurons of cortical microcircuits that may involve the role of dopaminergic, cholinergic, glutamatergic, and GABAergic systems [67], whereas elevated turnover of arachidonic acid signaling has been proposed as the pathophysiology underpinning the attenuated flush response to niacin in schizophrenia and its dynamic relationship to membrane polyunsaturated fatty acids [68]. Furthermore, future multiomics approaches might help clarify this and elucidate the vulnerabilities underlying FEP.

This study had limitations. First, our patients were not systematically ascertained; therefore, the study has limitations in generalizability. Second, FEP patients in various diagnostic categories were pooled in our analyses due to the instability of diagnostic categories over time in such patients [4, 5] and the substantial genetic overlap between schizophrenia and affective disorders [69, 70]. Third, different external samples of healthy controls were used to standardize each neurocognitive measure and NRA, which might result in biased estimates of effect size of different quantitative measures. Fourth, patients’ characteristics might systematically differ between FEP patients and schizophrenia patients of different familial loadings, although we used the identical external sample of healthy controls to standardize each neurocognitive measure and NRA to minimize the impact of these unknown differences. Last, despite our correction for multiple testing, the relationships between baseline measures and clinical features need future independent replication.

In conclusion, this FEP study of Taiwanese patients revealed the presence of neurocognitive performance and niacin response and their different relationships with clinical features, rendering this sample useful for future follow-up and incorporation of multiomics investigation.

### Electronic supplementary material

Below is the link to the electronic supplementary material.

Supplementary Material 1

## Data Availability

The datasets used and analyzed in the current study are not publicly available due to conditions in the participant consent and other ethical restrictions. However, the data that support the findings of this study are available from the corresponding author upon reasonable request.

## Abbreviations

FEP: First-episode psychosis
PANSS: Positive and Negative Syndrome Scale
PSP: Personal and Social Performance
CPT: Continuous Performance Test
WCST: Wisconsin Card Sorting Test
NRA: Niacin response abnormality
MPAs: Minor physical anomalies and craniofacial features
DSM-5: Diagnosis and Statistical Manual of Mental Disorders, Fifth Edition
VNR: Volumetric niacin response
SD: Standard deviation
