# Supplementary material for: Patients with first-episode psychosis in northern Taiwan: neurocognitive performance and niacin response profile in comparison with schizophrenia patients of different familial loadings and relationship with clinical features
Source: BMC Psychiatry. 2024 Feb 22;24:155. doi: 10.1186/s12888-024-05598-2 (PMC10885443; doi:10.1186/s12888-024-05598-2)
Supplement: Supplementary file 1 — Supplementary Material 1 [file 12888_2024_5598_MOESM1_ESM.docx]

**Supplementary information**

Table S1. Demographic characteristics and baseline diagnosis of patients with first episode psychosis across different sources of recruitment in northern Taiwan from 2016-2019.

Table S2. Baseline clinical characteristics and quantitative measures among patients with first episode psychosis stratified by recruitment source in Taiwan.

Table S3. Comparisons in the Z-score of five quantitative measures among patients with first episode psychosis, schizophrenia patients from simplex families, and schizophrenia patients from multiplex families.

Table S4. A multinomial logistic regression analysis of three groups of patients on neurocognitive measures.

Table S5. Spearman’s correlation of baseline clinical features and neurocognitive measures with the duration of untreated psychosis at baseline among patients with first-episode psychosis.

Table S6. Association between baseline measures and PANSS positive subscales at baseline with adjustment for potential confounders among patients with first-episode psychosis.

Fig. S1. The distribution of severity of each item from the PANSS

Fig. S2. Percentage of skin flush response at 3 time points for 2 niacin concentrations.

Table S1. Demographic characteristics and baseline diagnosis of patients with first episode psychosis across different sources of recruitment in northern Taiwan from 2016-2019.

| Characteristic | Inpatient  (n = 33) | Outpatient  (n = 47) | Statistic and p-value |
| --- | --- | --- | --- |
| Sex, n |  |  | χ^2^ = 9.20, p = 0.65 |
| Male | 15 | 19 |  |
| Female | 18 | 28 |  |
| Age (year), mean (SD) | 26.3 (6.1) | 25.0 (5.8) | t = 0.97, p = 0.34 |
| Duration of untreated psychosis (days), mean (SD) | 101.7 (128.8) | 154.2 (139.6) | t = -1.71, p =0.090 |
| Family history, n | 2 | 7 | χ^2^ = 1.52, p = 0.22 |
| Current smoking, n | 3 | 4 | χ^2^ = 0.01, p = 0.93 |
| Educational level, n |  |  | χ^2^ = 0.51, p = 0.78 |
| ≤ Junior high | 1 | 3 |  |
| Senior high | 9 | 12 |  |
| ≥ College | 22 | 29 |  |
| Diagnosis, n |  |  | χ^2^ = 8.38, p = 0.02 |
| Schizophrenia | 21 (64%) | 15 (31%) |  |
| Other non-affective psychosis | 8 (24%) | 25 (53%) |  |
| Affective psychoses | 4 (12%) | 7 (15%) |  |

Table S2. Baseline clinical characteristics and quantitative measures among patients with first episode psychosis stratified by recruitment source in Taiwan.

| Clinical variables | Inpatient (n=33) | |  | Outpatient (n=47) | | Statistic and p-value |
| --- | --- | --- | --- | --- | --- | --- |
|  | Mean (SD) | |  | Mean (SD) | |  |
|  | Raw score | z score |  | Raw score | z score |  |
| PANSS score |  |  |  |  |  |  |
| Total | 67.7 (21.8) | - |  | 67.1 (24.2) | - | t = 0.1, p = 0.91 |
| Positive | 17.8 (6.7) | - |  | 16.1 (7.2) | - | t = 1.0, p = 0.30 |
| Negative | 16.6 (7.7) | - |  | 16.1 (7.0) | - | t = -0.9, p = 0.39 |
| General psychopathology | 35.2 (10.8) | - |  | 34.9 (12.9) | - | t = 0.1, p = 0.91 |
| PSP score | 57.0 (17.8) | - |  | 65.3 (15.5) | - | t = -2.1, p = 0.03 |
| CPT |  |  |  |  |  |  |
| Undegraded d' | 3.9 (1.0) | -1.0 (1.4)^a^ *** |  | 3.8 (0.9) | -1.1 (1.3)^a^ *** | t = 0.3, p = 0.80 |
| Degraded d' | 3.2 (1.2) | -0.9 (1.4)^a^ *** |  | 3.5 (1.3) | -0.6 (1.4)^a^ *** | t = -0.8, p = 0.43 |
| WCST |  |  |  |  |  |  |
| Perseverative errors | 27.0 (23.8) | 0.5 (1.5)^b^ *** |  | 23.8 (17.6) | 0.4 (1.1)^b^ *** | t = 0.6, p = 0.55 |
| Categories achieved | 5.2 (3.3) | -0.4 (1.1)^b^ *** |  | 5.4 (3.0) | -0.3 (1.0)^b^  ** | t = -0.3, p = 0.75 |
| Niacin VNR for 0.1M and 0.01M | 9.8 (3.2) | -2.4 (1.8)^c^ *** |  | 10.1 (4.0) | -2.2 (2.2)^c^ *** | t = -0.4, p = 0.71 |
| MPA |  |  |  |  |  |  |
| Facial width | 13.6 (0.9) | 0.7 (1.0)^d^ *** |  | 13.6 (0.9) | 0.7 (1.0)^d^ *** | t = 0.0, p = 0.99 |
| Lower facial height | 6.8 (0.6) | -0.3 (0.7)^d^ *** |  | 6.7 (0.7) | -0.3 (0.8)^d^ *** | t = 0.0, p = 0.96 |
| Mouth score | 1.4 (0.4) | -1.0 (0.7)^d^ *** |  | 0.4 (0.8) | -1.1 (0.7)^d^ *** | t = 0.5, p = 0.59 |

^*^p value < 0.05 and ^***^p value<0.001 versus the comparison group.

Abbreviations: CPT = Continuous Performance Test; WCST = Wisconsin Card Sorting Test; VNR = volumetric niacin response; MPA = Minor physical anomalies and craniofacial features.

Table S3. Comparisons in the Z-score of five quantitative measures among the 80 patients with first episode psychosis in this study and two external comparison groups (schizophrenia patients from simplex families and schizophrenia patients from multiplex families).

|  |  |  | External comparison groups | | |  | Group comparison: ANOVA | |
| --- | --- | --- | --- | --- | --- | --- | --- | --- |
|  | FEP patients  (N = 80)  (Group 1) |  | Schizophrenia patients of simplex families^a^  (Group 2) |  | Schizophrenia patients of multiplex families^b^  (Group 3) |  |  | Tukey post |
| Z-score | Mean (SE) |  | Mean (SE) |  | Mean (SE) |  | P | hoc comparisons |
| Continuous Performance Test (CPT) |  |  |  |  |  |  |  |  |
| Undegraded d' | -1.06 (0.16) |  | -1.92 (0.05) |  | -2.48 (0.06) |  | <0.0001 | 1>2, 1>3, 2>3 |
| Degraded d' | -0.70 (0.17) |  | -1.31 (0.05) |  | -2.54 (0.05) |  | <0.0001 | 1>2, 1>3, 2>3 |
| Wisconsin Card Sorting Test (WCST) |  |  |  |  |  |  |  |  |
| −Perseverative errors^a^ | -0.44 (0.15) |  | -1.29 (0.05) |  | -1.22 (0.06) |  | 0.0004 | 1>2, 1>3 |
| Categories achieved | -0.32 (0.12) |  | -0.92 (0.03) |  | -1.05 (0.02) |  | <0.0001 | 1>2, 1>3, 2>3 |
| Niacin response (NR) abnormality |  |  |  |  |  |  |  |  |
| Volumetric NR for 0.1 M and 0.01 M | -1.25 (0.14) |  | -0.34 (0.12) |  | -1.27 (0.08) |  | <0.0001 | 2>1, 2>3 |

^a^Including 1649 for CPT and WCST as well as 1866 for NRA from simplex family (Wang *et al*. Genes Brain Behav 2018;17:49-55)

^b^Including 1314 for CPT and WCST as well as 176 for NRA from multiplex family (Hwu *et al*. Am J Med Genet B Neuropsychiatr Genet 2005; 134B:30-36)

Table S4. A multinomial logistic regression analysis of three groups of patients on neurocognitive measures.

| Z score | All |  | Simplex patients |  | FEP | | |  | Multiplex patients | | |
| --- | --- | --- | --- | --- | --- | --- | --- | --- | --- | --- | --- |
|  | Mean (SD) |  | Mean (SD) |  | Mean (SD) | OR | (95% CI) |  | Mean (SD) | OR | 95% CI |
| Continuous Performance Test |  |  |  |  |  |  |  |  |  |  |  |
| −Undegraded d'^a^ | 2.02 (1.97) |  | 1.80 (1.88) |  | 1.04 (1.35) | 0.55 | (0.64-0.90) |  | 2.39 (2.05) | 1.17 | (1.12-1.22) |
| −Degraded d'^a^ | 1.77 (1.73) |  | 1.27 (1.67) |  | 0.70 (1.41) | 0.78 | (0.66-0.93) |  | 2.51 (1.55) | 1.58 | (1.49-1.67) |
| Wisconsin Card Sorting Test |  |  |  |  |  |  |  |  |  |  |  |
| Perseverative errors | 1.25 (1.76) |  | 1.31 (1.72) |  | 0.36 (1.22) | 0.68 | (0.56-0.82) |  | 1.23 (1.83) | 0.97 | (0.93-1.02) |
| −Categories achieved^a^ | 0.94 (0.88) |  | 0.90 (0.94) |  | 0.30 (1.05) | 0.55 | (0.44-0.70) |  | 1.04 (0.76) | 1.21 | (1.10-1.34) |
| Niacin response abnormality |  |  |  |  |  |  |  |  |  |  |  |
| −Volumetric NR for 0.1 M and 0.01 M^a^ | 1.01 (1.43) |  | 0.34 (1.41) |  | 1.25 (1.16) | 1.68 | (1.35-2.08) |  | 1.27 (1.39) | 1.69 | (1.44-1.98) |

^a^Transformed to a negative value to let a more positive Z-score represent a greater impairment compared to the comparison group and hence denoted as −Undegraded d', −Degraded d' and −Categories achieved; −Volumetric NR for 0.1 M and 0.01 M.

Table S5. Spearman’s correlation of baseline clinical features and neurocognitive measures with the duration of untreated psychosis at baseline among patients with first-episode psychosis.

| Z-scores | CPZ equivalents  *r* (*P* value) | DUP  r (P value) | Age  r (P value) | Sex  t (P value) | Recruitment source  t (P value) | Family history  t (P value) | Current smoking  t (P value) | Educational level  F (P value) |
| --- | --- | --- | --- | --- | --- | --- | --- | --- |
| ***Clinical features*** |  |  |  |  |  |  |  |  |
| Positive and Negative Syndrome Scale score |  |  |  |  |  |  |  |  |
| Positive | 0.26 (0.02) | 0.09 (0.47) | 0.03 (0.80) | 1.56 (0.12) | 1.04 (0.30) | −0.19 (0.85) | −2.20 (0.03) | 2.11 (0.13) |
| Negative | 0.18 (0.12) | 0.21 (0.08) | −0.21 (0.07) | 1.72 (0.09) | −0.86 (0.39) | 0.46 (0.64) | 0.05 (0.96) | 0.05 (0.05) |
| Personal and Social Performance score | −0.22 (0.06) | 0.21 (0.06) | 0.10 (0.39) | −0.85 (0.40) | −2.14 (0.04) | −0.87 (0.39) | 0.75 (0.46) | 0.75 (0.46) |
| ***Quantitative measures*** |  |  |  |  |  |  |  |  |
| Continuous Performance Test |  |  |  |  |  |  |  |  |
| Undegraded d' | −0.18 (0.15) | −0.15 (0.22) | 0.19 (0.16) | −1.41 (0.16) | 0.25 (0.80) | 0.79 (0.46) | −0.94 (0.34) | 2.36 (0.10) |
| Degraded d' | −0.26 (0.03) | −0.08 (0.51) | 0.20 (0.11) | −1.37 (0.18) | −0.79 (0.43) | 0.91 (0.40) | 0.03 (0.98) | 2.28 (0.11) |
| Wisconsin Card Sorting Test |  |  |  |  |  |  |  |  |
| −Perseverative errors^a^ | −0.16 (0.18) | −0.11 (0.37) | −0.16 (0.33) | −0.90 (0.38) | −0.61 (0.55) | 0.77 (0.44) | −2.56 (0.02) | 1.18 (0.31) |
| Categories achieved | −0.14 (0.23) | −0.13 (0.28) | −0.09 (0.46) | −1.20 (0.23) | −0.32 (0.75) | 0.59 (0.56) | −2.06 (0.05) | 0.25 (0.78) |
| Niacin response (NR) abnormality |  |  |  |  |  |  |  |  |
| Volumetric NR for 0.1 M and 0.01 M | 0.19 (0.11) | 0.05 (0.69) | −0.06 (0.63) | 1.78 (0.08) | −0.36 (0.72) | −4.92 (<0.001) | −0.70 (0.49) | 0.84 (0.44) |
| Minor physical anomalies and craniofacial features |  |  |  |  |  |  |  |  |
| Facial width | 0.14 (0.26) | 0.10 (0.40) | 0.33 (0.01) | 5.44 (<0.001) | 0.01 (0.99) | −0.64 (0.52) | −1.09 (0.28) | 0.50 (0.61) |
| Lower facial height | 0.02 (0.89) | 0.00 (0.98) | 0.16 (0.18) | 3.20 (<0.001) | 0.05 (0.96) | 0.11 (0.91) | −0.59 (0.56) | 1.28 (0.28) |
| Mouth score | 0.01 (0.95) | −0.01 (0.89) | −0.13 (0.26) | 1.38 (0.17) | 0.55 (0.59) | 0.33 (0.74) | −0.42 (0.68) | 0.42 (0.94) |

^a^Transformed to a negative value to let a more negative Z-score represent a greater impairment compared to the comparison group and hence denoted as −perseverative errors.

Abbreviations: CPZ = chlorpromazine; DUP = duration of untreated psychosis.

Table S6. Association between baseline measures and PANSS positive subscales at baseline with adjustment for potential confounders among patients with first-episode psychosis.

|  | PANSS positive subscales | |
| --- | --- | --- |
| Z-scores | Adjusted for CPZ equivalents  β (P value) | Adjusted for current smoking status  β (P value) |
| Continuous Performance Test |  |  |
| Undegraded d' | −0.77 (0.23) | −1.29 (0.04) |
| Degraded d' | −0.19 (0.78) | −0.64 (0.34) |
| Wisconsin Card Sorting Test |  |  |
| −Perseverative errors^a^ | −1.06 (0.11) | −1.67 (0.01) |
| Categories achieved | −1.90 (0.01) | −2.42 (0.002) |
| Niacin response (NR) abnormality |  |  |
| Volumetric NR for 0.1 M and 0.01 M | 0.70 (0.09) | 0.82 (0.05) |
| Minor physical anomalies and craniofacial features |  |  |
| Facial width | 0.06 (0.94) | −0.14 (0.88) |
| Lower facial height | −0.13 (0.91) | −0.21 (0.85) |
| Mouth score | 0.06 (0.96) | −0.29 (0.82) |

^a^Transformed to a negative value to let a more negative Z-score represent a greater impairment compared to the comparison group and hence denoted as −perseverative errors.

Abbreviations: PANSS = Positive and Negative Syndrome Scale; NR = niacin response; CPZ = chlorpromazine.

Fig. S1. The distribution of severity of each item from the Positive and Negative Syndrome Scale (PANSS).

**
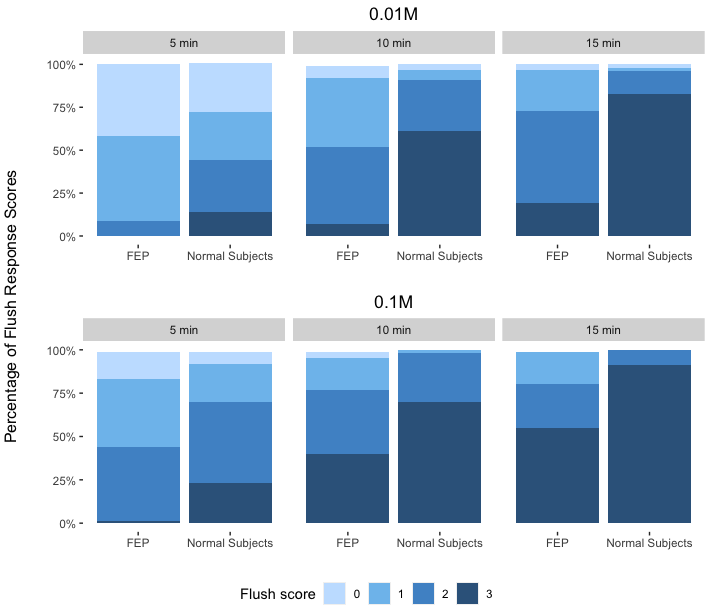
**

Fig. S2. Percentage of skin flush response at 3 time points for 2 niacin concentrations. FEP = first-episode psychosis.
